# Etanercept in juvenile idiopathic arthritis: Who will benefit?

**DOI:** 10.1186/1546-0096-9-S1-O28

**Published:** 2011-09-14

**Authors:** MH Otten, FHM Prince, W Armbrust, R ten Cate, EPAH Hoppenreijs, M Twilt, Y Koopman-Keemink, SL Gorter, KM Dolman, JF Swart, JM van den Berg, NM Wulffraat, MAJ van Rossum, LWA van Suijlekom-Smit

**Affiliations:** 1 Department of Pediatrics/Pediatric Rheumatology, Rotterdam, The Netherlands; 2 Department of Pediatrics/Pediatric Rheumatology, Groningen, The Netherlands; 3 Department of Pediatrics/Pediatric Rheumatology, Leiden, The Netherlands; 4 Department of Pediatrics/Pediatric Rheumatology, Nijmegen, The Netherlands; 5 Department of Pediatrics/Pediatric Rheumatology, The Hague, The Netherlands; 6 Department of Pediatrics/Pediatric Rheumatology, Maastricht, The Netherlands; 7 Department of Pediatrics/Pediatric Rheumatology, Amsterdam, The Netherlands; 8 Department of Pediatrics/Pediatric Rheumatology, Utrecht, The Netherlands

## Background

The pharmacological treatment approach for juvenile idiopathic arthritis (JIA) has changed substantially since the introduction of biologicals, with nowadays inactive disease as realistic treatment goal.

## Aim

To identify factors at baseline which predict etanercept treatment response and subsequently optimize treatment strategies.

## Methods

The Arthritis and Biologicals in Children Register (observational study, ongoing since 1999), includes all Dutch JIA-patients who used etanercept. Disease activity variables were retrieved prospectively at start of treatment, after 3 months, and yearly thereafter.

## Results

262 previously biologic-naive JIA-patients initiated etanercept; 71% female, 18% systemic-onset subtype. Median age at onset 6.9 (IQR 3.6-11.1) years, median follow-up 35.6 (IQR 17.4-53.6) months. In the long-term, the overall majority responded to etanercept and up to 40% reached inactive disease. Excellent response after 15 months (85 patients, 32%) was associated with low baseline disability (OR 0.49/point increase, 95%CI 0.33-0.74), fewer DMARDs used before etanercept (OR 0.64/DMARD used, 95%CI 0.43-0.95) and younger age at onset (OR 0.92/year, 95%CI 0.84-0.99); poor response (88 patients, 34%) was associated with female gender (OR 2.12, 95%CI 1.11-4.08) and systemic-onset subtype (OR 3.24, 95%CI 1.39-7.56). However, 24% of systemic-onset patients reached excellent response. Reasons for discontinuation: ineffectiveness in 78, adverse events (AEs) in 25, remission in 39 patients. Etanercept was well tolerated. Patients who developed AEs could not be identified at baseline.

## Conclusions

Excellent response was associated with baseline low disability and less DMARD-use before etanercept. Therefore, the focus should be on strategies with early introduction of etanercept to improve outcomes for JIA. The role of etanercept for the systemic-onset subtype remains debatable.

